# Using a virtual reality game to train biofeedback-based regulation under stress conditions

**DOI:** 10.1111/psyp.14705

**Published:** 2024-10-09

**Authors:** Lucie Daniel-Watanabe, Benjamin Cook, Grace Leung, Marino Krstulović, Johanna Finnemann, Toby Woolley, Craig Powell, Paul Fletcher

**Affiliations:** 1 Department of Psychiatry, University of Cambridge, Cambridge, UK; 2 Department of Physiology, Development and Neuroscience, University of Cambridge Ringgold Standard Institution, Cambridge, UK; 3 Ninja Theory Ltd, Cambridge, UK; 4 Department of Psychiatry, University of Cambridge, Cambridge, UK; 5 Cambridge and Peterborough NHS Foundation Trust, Cambridge, UK; 6 Wellcome Trust MRC Institute of Metabolic Science, University of Cambridge, Cambridge, UK

**Keywords:** anxiety, biofeedback, heart rate variability, interoception, respiration, stress

## Abstract

Physiological regulation strategies can be effective in reducing anxiety. However, while these strategies are often learned and practised under low-stress conditions, they are more likely to be required under conditions of high stress. We created virtual reality (VR) biofeedback games to both teach participants a breathing technique and then practise that technique under stress. We present two studies: the first provides a proof of concept, demonstrating that participants can apply the breathing technique during stress, with a significant lowering of both respiration rate and increase in heart rate variability (HRV) under stress (*p* <.001). The second study explicitly evaluated the effectiveness of training by comparing trained and untrained groups. Training was associated with a significantly greater HRV (*p* =.008) under stress. In within-group comparisons of HRV during stress compared to a baseline stressor presented before training, the trained group showed a significantly greater increase compared to untrained controls (*p* =.025). Our results show the feasibility and potential effectiveness of VR-based games for biofeedback training under experimentally applied stress. This may offer the opportunity for clinical techniques to more closely reflect the circumstances under which those techniques will be required.

## 1 Introduction

Optimal treatments for anxiety disorders combine pharmacological and psychological therapies (Bandelow et al., 2017) but these are limited in their efficacy and accessibility. This is important, given that anxiety disorders are the most common psychiatric disorders (Bandelow & Michaelis, 2015) and that anxiety frequently co-occurs with many psychiatric conditions, including autism and schizophrenia (Achim et al., 2011; Braga et al., 2013; Hollocks et al., 2019; White et al., 2009). Anxiety also has an appreciable impact outside the clinical domain (Kwong et al., 2021; Slee et al., 2019). The need for effective, accessible interventions is therefore pressing.

There is growing recognition of the importance of viewing psychiatric conditions and their symptoms in the context of the whole body, rather than focusing solely on the brain. Work examining interoception—the experience of internal bodily sensations (Craig, 2003)—is particularly relevant to anxiety researchers, given that anxiety often entails bodily experiences (Clark & Watson, 1991). There is a large body of evidence that physiological regulation, such as paced breathing, can lead to reductions in anxiety and improvements in emotional well-being (Chen et al., 2017; Doll et al., 2016; Kim et al., 2015; Mather & Thayer, 2018; Zaccaro et al., 2018). This informs various treatment options such as breath-focused mindfulness, which may be used in mindfulness-based cognitive therapies (Arch & Craske, 2006; Gu et al., 2015).

One promising approach to encouraging and facilitating physiological regulation is the use of biofeedback. Biofeedback provides individuals with explicit representations of physiological measures enabling them to recognize and regulate bodily responses. Biofeedback can include cardiac, respiratory, neural, electromyographic, and electrodermal regulation (Schoenberg & David, 2014) and has shown promising results in the treatment of several psychiatric disorders. While the most common regulation target has been the EEG signal (Schoenberg & David, 2014), cardiac-based measures, such as heart rate (HR) and heart rate variability (HRV; Ge et al., 2022; Goessl et al., 2017; Lehrer & Gevirtz, 2014; Weibel et al., 2023), also form an important target for biofeedback control. HRV refers to the variability in time between successive heartbeats within a given time period and it is a measure that has been associated with better physical (Kleiger et al., 2005) and psychological (Zaccaro et al., 2018) health. It is typically quantified using a variety of measures in the time and frequency domain (for a full review, see Shaffer & Ginsberg, 2017) and may provide a useful marker for stress and therefore a useful index of the degree to which a biofeedback intervention is effective in modulating stress responses. In our studies, we employed slow-paced breathing, which is known to enhance HRV through a variety of mechanisms that are under parasympathetic control (for review, see Lehrer & Gevirtz, 2014; Sevoz-Couche & Laborde, 2022).

Importantly, biofeedback-based regulation offers promise both alone (Lehrer et al., 2013) or in association with other psychological or pharmacological treatments as a means of managing anxiety. As well as equipping a person with skills that they can learn and deploy independently, it can be made accessible to those waiting for treatment access. However, it involves applications of techniques that demand learning and practice, requiring a high degree of engagement and motivation. Low motivation for treatment has been identified as one of the factors that can lead to patient nonadherence to treatment plans (Taylor et al., 2012). Therefore, combining biofeedback and breathing techniques into inherently motivational contexts is important. Previous studies using virtual reality (VR) biofeedback have reported both anxiety- and stress-reducing effects of these interventions. In addition, VR biofeedback may have advantages over traditional biofeedback due to increased participant motivation and attention (Lüddecke & Felnhofer, 2022). Blum et al. (2020) reported that, when comparing VR biofeedback to standard biofeed-back protocols, the VR group demonstrated increased self-efficacy and mindfulness compared to the standard biofeedback group.

One further potential advantage is that VR provides a convenient setting in which to provide rich and powerful experiences. This may be important in that both biofeedback and physiological regulation training (i.e., breathing exercises) are often done in a controlled and relatively relaxed setting, which may differ appreciably from the highly stressful circumstances in which the techniques are most pressingly required. This may make it difficult to generalize trained biofeedback-based regulation to its real-life deployment. Given this, the combination of video games, which are inherently motivational and enjoyable ways to develop skills (Granic et al., 2014) with VR, which provides immersive lifelike experiences, including stressful ones, offers new possibilities. Recently, researchers have developed a variety of games with the aim of teaching players emotional regulation skills (Cheng & Ebrahimi, 2023; Gummidela et al., 2021; Schuurmans et al., 2018; Weerdmeester et al., 2022, among others).

Here we describe two studies conducted to determine whether a VR-based game intervention would allow healthy participants to successfully deploy a prelearned breath control strategy in the context of a stressful experience. Each study used two VR game experiences in collaboration with Ninja Theory Ltd., a Cambridge-based video game design studio (www.ninjatheory.com).

Study 1 determined the feasibility and acceptability of applying the breathing technique in the context of a moderate stressor. It examined physiological and subjective responses to both the respiratory training and the stressful experience. Participants initially underwent baseline cardiorespiratory measures before receiving a 5-min VR breath-training game in a relaxed setting. Following this, they were immersed in a 10-min stressful biofeedback experience in which the successful completion of the game was dependent on their ability to maintain control of their heart rate using the newly learned breathing technique. Our primary question was whether people would be able to deploy the breathing pattern, and this was assessed by comparing their breathing rate during the stress experience with that measured during the initial baseline assessment. Our secondary question was whether participants’ ability to control their breathing under stress would modify physiological (HR and HRV) and subjective markers of stress.

Study 2 used a between-group design to more formally characterize the impact of breath-regulation training. A trained group received four sessions of breathing control training over two visits (5–7 days between visits). The control group received no training. We also included a different stressful VR experience (involving a monstrous home intruder) prior to any training in order to index baseline stress reactivity.

## 2 Study 1: Proof of Concept To Assess Feasibility and Acceptability of Applying Breath Control Under VR Stress

### 2.1 Materials and methods

Ethical approval for this study was obtained from the Cambridge Psychological Research Ethics Committee, reference number PRE.2021.094.

#### 2.1.1 Participants

Participants were recruited through advertisement in the Cambridge area. We included healthy participants aged 18–40 years with no self-reported significant history of neurological, psychiatric, cardiovascular, or metabolic disease or of recent alcohol or substance misuse. While a history of anxiety or depression was not an exclusion factor, we did not recruit participants on psychoactive medication or on drugs affecting cardiovascular function. Where there was uncertainty about the participant’s suitability, a clinical member of the study team reviewed the participant’s responses to screening questions before proceeding. Apart from a need for normal or corrected-to-normal (with glasses or contact lenses) vision we used no exclusion criteria specific to VR. However, participants were instructed that should any concerns arise, such as headset discomfort, motion sickness, or headache, they should remove the headset and we would stop the study. We also excluded pregnant participants.

In total, 53 participants took part in this study. Five participants were excluded due to noisy pulse plethysmography (PPG) data. Four participants were excluded due to incomplete data collection resulting from equipment errors. PPG data were considered excessively noisy if over 1 min worth of data in totally showed unreliable peak detection, or if more than three independent areas of the PPG trace needed to be removed due to incorrect peak detection. No participants were asked to stop the experiment due to the negative effects of the VR setup (e.g., dizziness).

Respiration data were only available for a subset of participants due to technical problems (lack of appropriate respiration band sizing and noisy data). Noisy data were identified as data where respiratory peaks were not identifiable for >1 min worth of data. Full respiration data sets were available for 29 participants. Twenty-six participants had both full respiration and PPG data.

#### 2.1.2 Study procedure

Participants attended the lab on one occasion for approximately 1 h upon arrival, they were given detailed information about the study and offered the opportunity to ask questions before giving their informed consent to participate. Participants were then asked to complete a series of questionnaires relating to their mental health, including the State-Trait Anxiety Inventory (Spielberger, 2012), Beck Depression Inventory-II (Beck et al., 2011), and Multimodal Assessment of Interoceptive Awareness (Mehling et al., 2012). Alongside this, they were asked to report how often they played video games, as well as if they had previous experience of VR, and how much they enjoyed watching horror films.

#### 2.1.3 Baseline

After completing these questionnaire measures, participants were taken to the VR section of the study. The testing environment was isolated to minimize extraneous noise and, during subsequent experimental procedures participants were wearing the VR headset with incorporated headphones to avoid noise other than those that were part of the experiment. Firstly, they were connected to the BioRadio™ (Great Lakes NeuroTechnologies), a multichannel physiological measurement device. The BioRadio was set to record cardiac activity through pulse PPG via a fingertip sensor on the index finger of the nondominant hand, and respiration via a resistance belt placed around their chest (at a sampling rate of 75 Hz). Their hands were supported on a conveniently positioned table and care was taken to avoid pressure on the PPG sensor. A 5-min baseline recording, with participants sitting quietly, was obtained before going on to the two different VR experiences.

#### 2.1.4 Virtual reality setup

After the baseline measurement, participants were asked to put on the VR headset. The headset used for this study was the Vive Pro Eye (HTC). Participants were informed at the beginning of the VR section of the study that they were free to stop at any time and to inform the experimenter if they experienced any negative effects from the VR (such as motion sickness). Both VR games were undertaken with participants seated, with the stressor game beginning immediately after the end of the training.

#### 2.1.5 Training game

The aim of the training game was to teach participants a breathing technique. In this game, participants found themselves seated on a boat on a calm sea. They were instructed that the boat was being driven by their own biometrics and told that, to move the boat forward, they must try and lower their heart rate by practicing a paced breathing exercise structured around a 5-5-5 breathing pattern. Participants were then prompted to practise the paced breathing exercise by following onscreen prompts, saying “inhale,” “hold,” and “exhale” at fixed 5-s intervals each. This breathing pattern aimed to help participants achieve a slow deep breathing rate of 4 breaths per minute. This session lasted last from 4 to 7 min, with a mean completion time of 307 s (range 240–374 s, standard deviation 26 s). The length of each session was determined by the speed of the boat which in turn was dependent on successful adherence to the breathing pattern.

**Figure 1:**
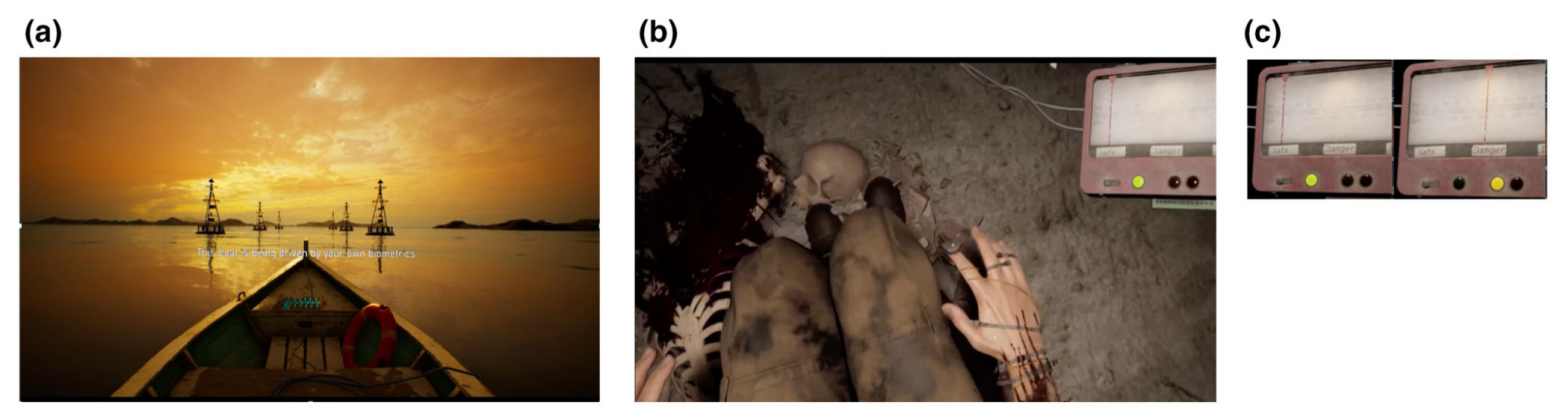
Ex(a) Example still from the training session. Participants’ HR trace was fed back to them (cyan trace in the prow of the boat), and they also received both written and verbal instructions on the breath regimen. Further ambient sounds of the water were presented to encourage relaxation. (b) Example still from the stressor session. In this still, the participant is looking down and can see a representation of their body including the attachment of the pulse device to their right index finger. The stress monitor (top right) was experienced as being fixed to their headset so that it was always in their field of view no matter where they looked. In the image, their stress index (see main text) is within the “safe zone” (with the indicator to the left of the display and the green light on). When aroused it could move to the “danger” or “death” zones. (c) Example of the biofeedback mechanism implemented in the stressor session demonstrating the difference between the “safe” zone in green and the “danger” zone in amber, with the red vertical dial indicating the continuous change in stress score.

The training comprised two different visual prompts concerning the participants’ physiological signals. First, their PPG waveform was displayed on the bow of the boat (see Figure 1). Second, the light particles in the air expanded during the “inhale” prompt and contracted during the “exhale” prompt.

While the original plan had been to relate the speed of the boat inversely to the participant’s heart rate, a coding error meant that boat speed was positively correlated with HR. The speed of the boat was determined using arbitrary units in which 1 beat increase in HR led to a 1 unit change of speed, with a constant of 50 units added to the participant’s average HR. The 50-unit constant was added as the boat movement when tested at 70 units was imperceptibly slow. In practice, we believe that this coding error had a negligible effect since participants had no reference for the speed of the boat and being focused on the verbal and visual breathing instructions, were unlikely to be aware of any changes in heart rate.

#### 2.1.6 Stressor game

After the training session, participants began the stressor game. Participants were briefed in advance that this virtual experience involves entering a dungeon. The goal of the game is to avoid detection by a blindfolded creature who could hear their heartbeats very well and hence had a high chance of discovering them if their heart rate became too high. Upon detection by the creature, participants would lose the game, and the experience would be terminated. This experience lasted for a maximum of 10 min, with 9 participants not completing the entire experience (in other words, being detected and losing the game). The mean time spent in the stressor game for all participants was 549 s, and for the participants who were detected by the creature and lost the game, the mean time spent in the stressor session was 318 s.

After having completed the procedure (baseline, training, and stressor), participants were debriefed and asked how stressful they found the different training and stressor sessions on a 1–10 scale.

The biofeedback component of this game was communicated through a display on the top right of the screen showing three lights and a moving dial. The display was affixed to a headframe in the virtual scenario and therefore visible to the participants regardless of their head position or which direction they turned their head. The lights were green, amber, and red. These indicated the participants’ relative stress level, as indexed by their heart rate relative to the training session. Participants were instructed that the lights were indicative of their safety—how likely it was that they would be found by the creature—with green indicating that they were safe, amber indicating their chances of being found were rising, and red indicating that it was likely that they would be discovered. This calculation of the stress score was implemented in the following way:

$$C=2\text{Ln}(3)\frac{\text{RefBPM-BPM}}{\text{RangeBPM}},$$

$$\text{StressScore}=\frac{1}{e^{c}+1},$$

where RefBPM is the mean beats per minute (BPM) acquired during the training phase, and BPM is the current mean BPM over a 30-beat rolling window throughout the testing phase. RangeBPM was set to 25 for this study.

The stress score calculation was chosen to give a comparable score across participants, regardless of their baseline heart rate. It uses a sigmoid function to keep the stress score in a range from 0 to 1, with RangeBPM scaling the score such that RefBPM + RangeBPM provides a stress score of 0.9 (red), and RefBPM − RangeBPM gives a stress score of 0.1 (green). RangeBPM is a variable that can be manipulated by the experimenter to make the stressor easier or harder and can be set from 5 to 100 BPM (in increments of 5).

When the stress index computed by the above calculation reached a value of 0.41 then the light would change to amber, and when it reached a value of 0.778 the light would change to red. Being in the red zone during one of five prespecified time points at which participants could die would trigger a death sequence, thus ending the game early. Participants were instructed to try and continue the breathing technique that they had previously learned in the training session to keep their heart rate low and thus decrease the risk of “dying.”

The heart rate increase required for the red zone to be reached (RangeBPM) can be prespecified in the experimental setup. For this experiment, RangeBPM was set to 25. This value was chosen after piloting as a value that should lead to most participants completing the full experience of the stressor, allowing comparable data analysis while still making the experience challenging. Pilots (*n* = 5) were completed with RangeBPM = 15, which led to 3 pilot participants “dying” early and was thus considered too restrictive. Pilots were also completed with RangeBPM = 30 (*n* = 2), which led to pilots rarely leaving the “safe” green zone which was considered too easy. Twenty-five were chosen for this study instead of 20 to improve the likelihood of participants completing the entire session.

##### Analysis

Due to the use of PPG in this study, we technically measured pulse rate variability which, while not the same as HRV, is highly correlated to it (Bulte et al., 2011). We will henceforth refer to it as “HRV” as the more commonly understood term. The (HRV) metric used for this study was time domain measure SDNN—the standard deviation of N–N intervals (or the standard deviation of the interbeat interval of normal beats). We selected SDNN as our preferred metric for HRV given that, in relatively brief resting recordings, variation in SDNN is primarily driven by respiratory sinus arrhythmia (RSA) (Shaffer & Ginsberg, 2017), especially under conditions of slow-paced breathing. RSA is parasympathetically driven and therefore SDNN would provide an indirect marker for whether participants were able to regulate stress responses, particularly given the brief recording times and the focus on respiratory control in our study. Conversely, other time-domain measures such as root mean square of successive differences (RMSSD) may be less appropriate for paced breathing techniques given a less certain relationship with respiratory rate, leading to potential ambiguities (Shaffer & Ginsberg, 2017). Moreover, slow breathing has complex effects on measurements in the frequency domain, in particular increasing the low frequency (0.04–0.15 Hz) power as a consequence of reducing the frequency of respiration-influenced HRV. So, in keeping with recommendations (Chang et al., 2013; Hernando et al., 2016) frequency domain measures were not used. Thus, while no measure is without ambiguities and limitations, we reasoned that SDNN would be optimal under the current circumstances.

HR and HRV metrics were extracted using the Systole package (Legrand & Allen, 2022) in Python (version 3.10.5). Systole employs an automated correction algorithm that detects missing beats, long beats, and ectopic beats, which was implemented in the HRV detection. In addition to this correction, all HRV data were manually inspected, and if there were noisy periods in the PPG trace of >3 beats, these sections were manually removed. The SDNN measure was normalized for each participant according to their heart rate, using the cvSDNN adjustment suggested by De Geus et al. (2019). This is important since heart rate has a clear relationship with HRV: we would normally have expected increases in heart rate to be associated with reductions in HRV. Adjustment was made using the HR calculated using the HR measure from each of the different conditions (i.e., baseline SDNN was adjusted for baseline HR, training session SDNN was adjusted for training session HR, and biofeedback stressor SDNN was adjusted for biofeedback stressor HR). The adjustment for cvSDNN is as follows:

$$\text{cvSDNN}=100\times \frac{\text{SDNN}}{\text{IBI}},$$

where IBI is the mean interbeat interval for the session.

Respiration data were analyzed using MATLAB (version R2022b). Respiration data were first passed through a butterworth filter using the “butter” function in MATLAB. The butter filter used a 10th-order low-pass filter with a cutoff frequency of 0.1. A peak detection algorithm was implemented to determine the number of breath cycles per minute. All results were visually inspected by an experimenter to ensure the consistency of the outputs. After visual inspection, the experimenter was able to manually enter the number of any peaks detected or missed in the peak detection phase. From the respiration rate, an adherence score was calculated (4— breaths/min). This was to show how closely participants adhered to the breathing rate outlined in the training session (4 breaths per minute).

We analyzed the data according to the primary question concerning whether there was evidence that participants were able to deploy the breathing technique in the context of the VR stressor. We quantified the effect of training on breathing by comparing breathing rate before (during the initial baseline recording) and after (during dungeon stressor) the boat training experience. Adherence was indicated by a breathing rate during the stressor that was closer to the trained rate than it had been pre-training.

Our secondary analysis examined how HR and HRV during the stressor differed from these metrics during the initial baseline session. Finally, complementary exploratory analyses were carried out to determine whether those individuals showing greater levels of breathing adherence showed the greatest tendency to reduce the impact of the stressor on changes in HR and HRV. In addition, we sought to relate these effects to individual variations in stress experiences.

### 2.2 Results

All other analyses were conducted in R (version 4.3.1). The code is available at https://github.com/lucied-w/biofeedback-concept-paper.

### 2.3 Demographics

Of the 44 participants (after exclusions), mean age was 25 (range 18−39), with 21 female participants, 22 male participants, and 1 nonbinary or gender nonconforming participant.

The mean age was 25 years old (SD = 4.61). The mean BDI score was 5.67 (SD = 4.52), indicating low levels of depression. Mean trait anxiety scores were 33.97 (SD = 8.24), state anxiety 33.84 (SD = 9.41) indicating moderate levels of both trait and state anxiety.

### 2.4 Respiration data loss

Due to the limited number of participants for whom we had full respiration data, we examined whether there were demographic differences between the participants who we had respiration data for and who we did not have respiration data for (Table 1).

The participants without respiration data had a higher resting heart rate, with most other demographic metrics being similar between groups.

### 2.5 Subjective stress ratings

Participants were asked to subjectively rate how stressful they found both the training and the stress conditions, on a scale of 1–10. The mean stress rating for the training was 1.36, and the mean stress rating for the stressor was 5.61.

### 2.6 Cardiac and respiratory measures

**Figure 2:**
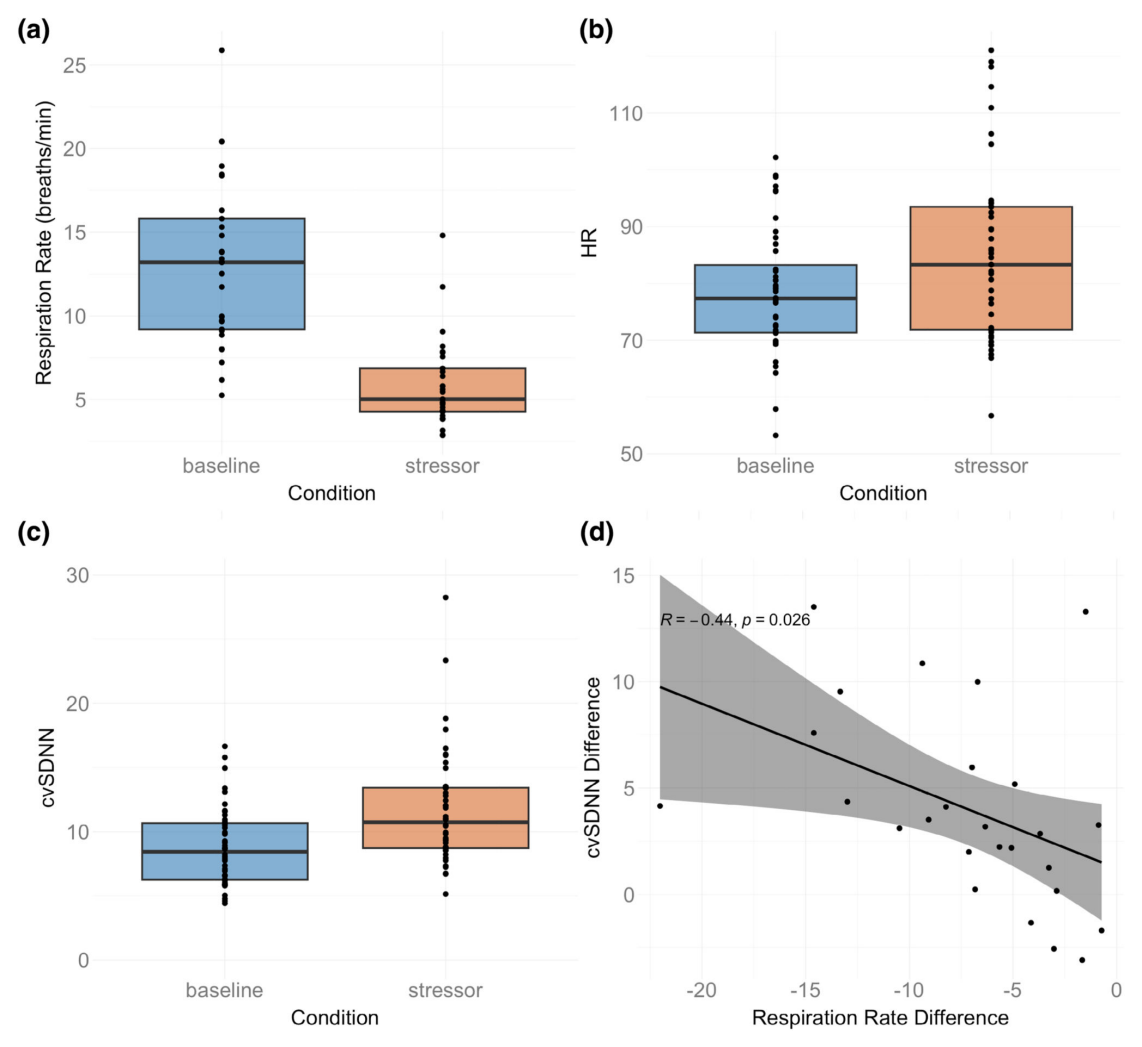
(a) Boxplot of the respiration rate for baseline and stressor conditions. (b) Baseline-normalized HR in baseline and stressor games. (c) Baseline-normalized cvSDNN in baseline and stressor games. (d) Correlation between the difference between the stressor and baseline (stressor − baseline) for cvSDNN and respiration rate.

The mean HR in the baseline condition was 78.49 bpm (SD = 11.00), in the training was 78.85 bpm (SD = 10.28), and in the stressor was 85.06 bpm (15.66). Mean cvSDNN in baseline was 8.79 (SD = 3.04), in the training was 11.68 (SD = 3.07), and in the stressor was 11.71 (SD = 4.47; see Figure 2).

For the subset of participants with respiration data, the mean in the baseline was 13.02 breaths/minute (SD = 4.92), the mean in the training was 4.27 breaths/minute (SD = 0.90), and the mean in the stressor was 5.90 breaths per minute (SD = 2.65; see Figure 2).

The respiration rate decreased for all 29 participants for whom we had respiration data, *t*(43) = 6.87, *p* <.0001, between the baseline and the stressor. The distribution of adherence scores to the breathing rate in the training game is available in the supplement (Figure S2).

There was an increase in heart rate between baseline and stressor. The mean heart rate in the stressor was 85.0, compared to 78.5 at baseline, *t*(43) = 03.35, *p* =.002. However, there was *also* an cvSDNN increase between baseline and stressor, indicating that there was an in-creased level of cardiac parasympathetic control—mean cvSDNN at baseline was 8.79 compared to 11.71 in the stressor, *t*(43) = −4.65, *p* <.0001. Comparison between cvSDNN at baseline, training, and stressor is available in the Supplementary Data (Figure S1).

We tested correlations between respiration and cardiac metrics. Here we describe correlations between baseline normalized respiration and cardiac measures, meaning the respiration rate during the stressor minus the respiration rate at baseline (Resp_stressor_ − Resp_baseline_) and the equivalent for the cvSDNN and HR values. These will be described as “baseline-normalized” values.

Correlations between baseline-normalized respiration and baseline-normalized cvSDNN showed a significant correlation, *r*(24) = −.44, *p* =.026 (see Figure 2). This suggests that participants showing the greatest tendency to reduce respiratory rate during the stressor (in line with training) showed an attenuation of the HRV marker for stress. However, correlations between baseline normalized respiration and baseline normalized HR were not significant.

#### 2.6.1 Summary of study 1: Proof of concept

To summarize, study 1 provides evidence that adherence to the trained breathing rate is possible in the context of a moderate stressor and that successfully doing so is associated with an elevation in HRV suggesting a reduced physiological stress response. We also show that the greatest positive impact on HRV was found in individuals with the greatest adherence to the breathing strategy. This provides proof of concept of the feasibility and potential effectiveness of the VR game-based approach. Study 2 below used a between-subjects design to demonstrate that the breathing and HRV changes are not nonspecific effects of time but are directly relatable to the training intervention.

## 3 Study 2: Controlled Trial

### 3.1 Materials and methods

Ethical approval for this study was gained from the Cambridge Psychological Ethics Research Committee, number PRE.2022.086.

#### 3.1.1 Participants

Inclusion and exclusion criteria were identical to Study 1.

Participants were recruited and assigned to either control or training group. Participants were screened before inclusion on the study and were sent a short online questionnaire asking their age, gender, and for them to complete the trait section of the STAI (Spielberger, 2012). Participants in the control and training groups were subsequently matched on these measures.

Both control and training groups were asked to attend the lab for two sessions, 5−7 days apart. The structure of the sessions was identical for control and training groups, with the exception that the training group were asked to play the training game twice in each session. The testing room setup was the same as in Study 1.

#### 3.1.4 Session 1

##### Control group

Upon arrival, participants were given details information about the study and offered the opportunity to ask questions before giving their informed consent to participate. They initially answered some baseline questionnaires: the Beck Depression Inventory (Beck et al., 2011), the State Trait Anxiety Inventory (Spielberger, 2012), and the Multimodal Assessment of Interoceptive Awareness (Mehling et al., 2012). After this, they were asked to take baseline physiological measurements. As in Study 1, participants’ physiological metrics were measured using a BioRadio (Great Lakes Neuro Technologies Ltd), using a respiratory belt, and pulse PPG via a fingertip sensor on the nondominant hand. In the first session, participants took a baseline of their physiological measurements, which was taken over a 5-min period with participants seated quietly.

Then participants were asked to put on the VR headset, which was also the Vive Pro Eye (HTC), for the “intruder” VR scenario. The intruder VR scenario was used as an index of participants’ physiological reactivity during Session 1. In this scenario, participants are seated at the base of a large staircase. The lights flicker and music plays from a radio. Following a flash of lightning there is a power failure, and the environment goes dark. A crash is heard from upstairs, indicating an intruder. Seated in the dark, participants hear, and see fleetingly through flashes of lightning, a monster progressing down the stairs towards them. This scenario is almost entirely passive, with the only interaction the participants have with the scenario being a brief period where they can move a virtual torch using the handheld controller. Otherwise, they have no control or input into the scenario.

This scenario differs from the dungeon as the dungeon involves active participation, as participants can exert control by regulating breathing, whereas the intruder experience is entirely passive. However, as these two scenarios have been shown to produce similar subjective levels of stress in our previous studies (with participants rating the stressfulness of the dungeon as 5.61/10 in Study 1, reported above, and participants (*n* = 47) rating the intruder stressor of 51/100 in a forth-coming paper), we used both as similar stress inductions to examine differences in physiological reactivity between the training and control group before and after training.

##### Training group

The training group’s Session 1 was identical to the control group, with the addition of playing the boat training game twice at the end of the session. These training games lasted on average 218 s. The shorter duration of the boat training game for Study 2 may be due to the participants having an elevated heart rate after the intruder VR experience, and therefore the boat moving faster.

#### 3.1.3 Session 2

##### Control group

In Session 2, the control group returned and were given information on the structure of the session by the experimenter. They were then attached to the BioRadio, as in Session 1. When attending for Session 2, the control group began by undergoing a repeat baseline physiological measurement. This was primarily to ensure that the structure of the session was comparable to that for the training group, who began the session with physiological measurements taken in a repeat of the boat training game (see below). After this, they were asked to do the biofeedback stressor VR, which is the same as described in Study 1. The only difference regarding the biofeedback stressor was that all participants completed the full game by turning off the possibility of early death—hence, the experience lasted for 10 min for all participants. The only other difference was that in Study 2, RangeBPM was set to 15 BPM. In previous piloting, RangeBPM set to 15 led to more time spent with the biofeedback monitor showing either amber or red lights, leading to a greater sense of urgency for participants to regulate their HR. By implementing this RangeBPM without the possibility for death, we aimed to increase the motivation for participants to self-regulate, while standardizing the time spent in the VR scenario.

After the end of the biofeedback stressor game, control participants were asked to fill in the same questionnaires that they had filled in at the beginning of Session 1. They were also asked to rate how stressful they found the bio-feedback experience. Afterward, they were debriefed, and the control group were offered the option of experiencing the boat training if they desired.

##### Training group

The training group’s Session 2 was the same as the control group, except they were given refresher training on the breathing strategy by going through the training game twice before being exposed to the biofeedback stressor game.

##### Analysis

The extraction of HR, HRV metrics, and respiration metrics were the same as in Study 1. As in Study 1, SDNN was normalized for each participant according to their heart rate, using the cvSDNN adjustment suggested by De Geus et al. (2019).

Data were baseline-normalized, meaning that the baseline values for the physiological measures were subtracted from target values. For example, to baseline-normalized HR in the biofeedback stressor = HR_stressor_ − HR_baseline_. All results reported here are baseline-normalized.

### 3.2 Results

Respiratory analyses were conducted in MATLAB (version 2022b). Analyses of the PPG data were conducted using the Systole package in Python (version 3.10.5). All high-level analyses were conducted in R (version 4.3.1). The code is available at https://github.com/lucied-w/two-arm-biofeedback.

### 3.3 Demographics

In total, 71 participants took part in this study: 35 in the control and 37 in the training group. Five participants from the control group and seven participants from the training group were discounted out due to failure or inability to attend the second session, leaving 30 participants in each group. During analysis, two participants were excluded from the training group due to noisy PPG data (with the same exclusion criteria as Study 1), and three from the control group. For subsequent analyses, we report 27 participants in each group for the cardiac data, having excluded one participant at random from the training group to ensure equal group size.

In the control group, 7 sets of respiration data were lost: 4 due to noise and 3 due to equipment connection errors. In the training group, 2 sets of respiration data were lost due to noise. This left us with 23 complete respiratory data sets for the control group and 28 in the training group. To ensure equal group size, 5 participants were randomly excluded from the training group.

Participants were matched for age, gender, and trait anxiety during recruitment. After exclusions, both groups comprised of 13 males, 13 females, and 1 nonbinary or otherwise nonconforming participant. The mean age for the control group was 25 (SD = 5.46), and the mean age for the training group was 24 (SD = 5.47). The mean trait anxiety score for the control group at recruitment was 38.00 (SD = 8.62), and the mean for the training group was 37.60 (SD = 8.40).

### 3.4 Differences in cardiac and respiratory measures in the stressor

Paired *t* tests were performed to examine the differences in baseline-normalized HR, cvSNN, and respiration rate in the control and training groups during the biofeedback stressor game.

**Figure 3:**
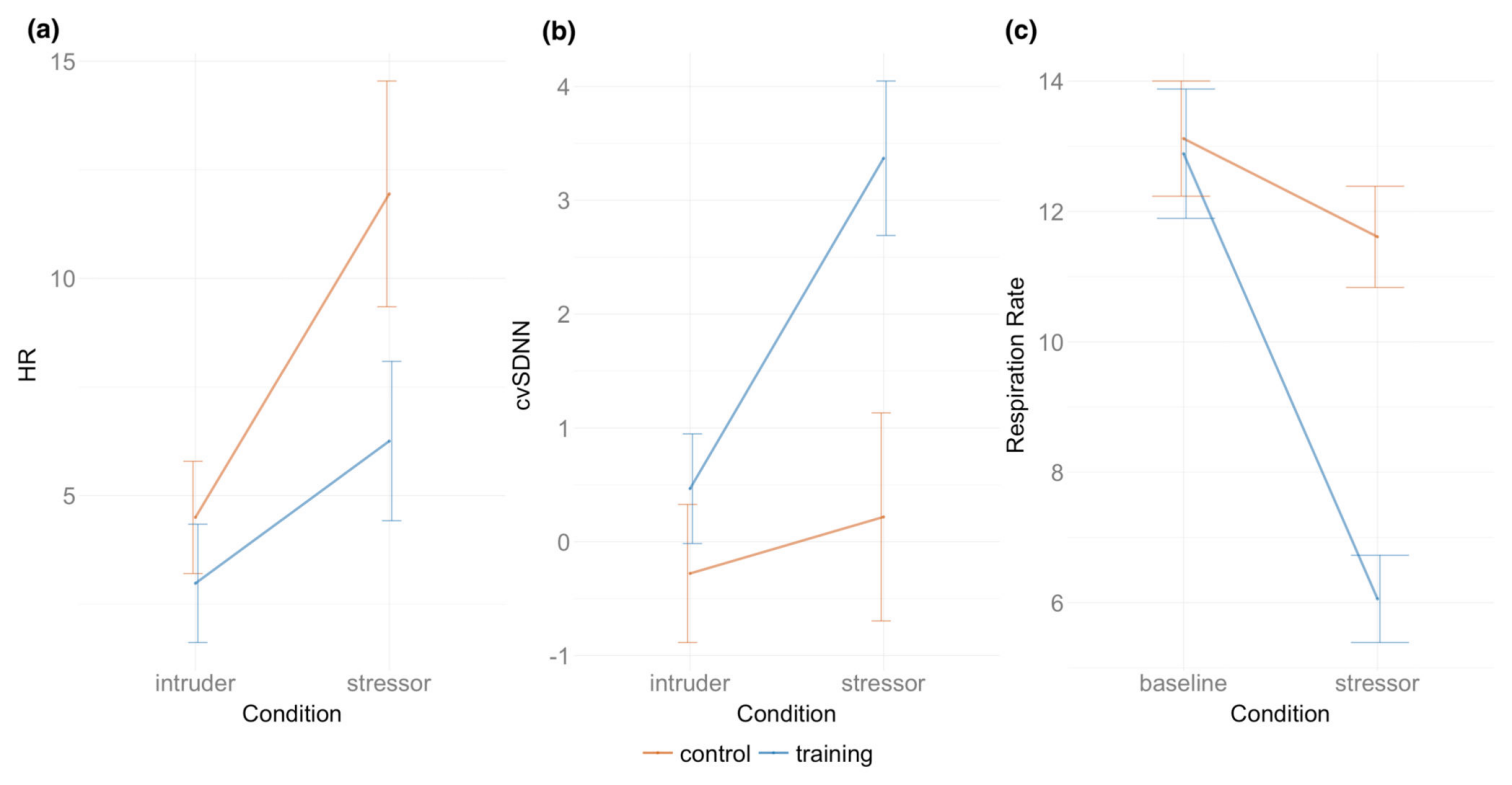
(NB “Intruder” refers to the initial pretraining stressor in Session 1 involving an empty house where an intruder breaks in) (a) Baseline-normalized HR in intruder and biofeedback stressor scenarios in control and training groups with standard error bars. (b) Baseline-normalized cvSDNN in the control and training group in the intruder and biofeedback stressor scenarios with standard error bars. (c) Respiration rate at baseline and in the stressor game for the control and training groups with standard error bars.

Baseline normalized HR showed no significant difference between control (11.94 BPM) and training (6.26 BPM) groups, *T* = −1.78, *p* =.08. Baseline normalized cvSDNN did show a significant difference, with the training group (3.37) showing higher cvSDNN than the control (0.22), *T* = 2.77, *p* =.008. Baseline normalized respiration rate was also significant, with the training group showing a greater decrease in respiration (−6.83 breaths/minute) compared to the control group (−1.50 breaths/minute), *T* = 5.27, *p* <.001 (see Figure 3).

### 3.5 Two-way ANOVA

We compared the physiological changes in Session 1 intruder scenario and Session 2 biofeedback stressor game. A two-way repeated measures ANOVA of the HR data showed a significant main effect of time, *F*(1, 50) = 12.368, *p* <.001, with both control and training groups showing a greater HR in the main (dungeon: control = 86.96; training = 82.41) stressor than in the initial baseline (intruder: control = 79.51; training = 79.14) stressor.

The two-way repeated measures ANOVA of the cvSDNN data showed a significant difference in group *F*(1, 50) = 8.13, *p* =.006, time *F*(1, 50) = 4.80, *p* =.033, and an interaction in group × time, *F*(1, 50) = 5.330, *p* =.025. Post hoc analyses found that the dungeon had a higher mean cvSDNN (5.55) overall than the intruder scenario (−3.38), *p* =.003. The training group also had a higher cvSDNN (10.36) than the control group (−8.19), *p* =.001. The interaction effect demonstrated that the training group showed a higher cvSDNN in the biofeedback stressor over the intruder scenario compared to the control group (see Figure 3, *p* =.001).

### 3.6 Questionnaire results

Participants were asked about their subjective levels of stress during the scenarios. Participants in the training group reported finding the dungeon experience significantly more stressful than the participants in the control group. Training group participants reported a mean level of 6.8, whereas the control group reported a mean level of 5.4, *t* = 2.84, *p* =.006.

### 3.7 Pooled studies questionnaire results

We combined the questionnaire results from Study 1 and Study 2 (training group only) to see if this larger sample produced any correlations with the self-report questionnaires. We found no significant correlations with the physiological results reported.

## 4 Discussion

We report two studies: Study 1 established the feasibility and acceptability of using a stressful VR experience as a setting in which to employ a recently learned breathing control technique to manage physiological responses through biofeedback. It further provided preliminary evidence that the training was associated with successful breathing control and a corresponding increase in HRV. However, the latter finding requires comparison to an untrained control group, and Study 2 therefore used a between-group design, demonstrating that participants who underwent training showed increased HRV and decreased respiration compared to untrained participants. Unexpectedly, training was associated with an increase in subjectively reported stress, which we discuss below.

The impact of slow-paced breathing (SPB) on HRV is of potential value both clinically and nonclinically. However, it is important to acknowledge that the mechanistic underpinnings of this effect are highly complex, resulting from several interacting factors reflecting the central and peripheral effects of respiratory function on both blood pressure and HR (Sevoz-Couche & Laborde, 2022). While the optimal frequency and pattern of SPB remains unclear, 0.1 Hz breathing (5 s inspiration; 5 s expiration) may produce maximal effects on HRV. It has also been suggested that a delay (of greater than 1 s) between the two phases may further optimize the impact on physiological markers of stress (Sevoz-Couche & Laborde, 2022). However, the latter remains unclear and further consideration is outside the scope of this paper. Our own choice of breathing rate (0.07 Hz: 5 s inspiration, 5 s hold, 5 s expiration) is slightly slower than the 0.1 Hz favored by Sevoz-Couche and Laborde (2022) but, nonetheless, had a significant impact on HRV in the anticipated direction.

The first of our two studies examined both the adherence to a prelearned breathing rate when subjected to a moderate degree of stress and the degree to which this adherence was associated with an elevation in HRV. HRV increased compared to a baseline measurement after respiration training. It was also demonstrated that those individuals showing the greatest adherence to the trained breathing rate showed the greatest increase in HRV. However, the design of Study 1 is not able to provide evidence that the training (rather than a nonspecific time effect) was causally related to the physiological changes during ensuing the stress experience. Notwithstanding this, the slowed respiratory rate provides a plausible explanation for the increase in HRV given the well-known mechanisms described above (Sevoz-Couche & Laborde, 2022).

Study 2 was designed to control for nonspecific effects by including a matched, untrained group of participants. We demonstrated a significantly greater HRV in the trained compared to the untrained group during the VR biofeed-back stressor game. Moreover, the trained group showed a significance increase in HRV when comparing a pretraining stress (intruder experience) to the posttraining bio-feedback stress game. This was seen as a group-by-session interaction demonstrating the change from baseline in the trained group was significantly greater than the change in the untrained group. While we did not collect subjective stress ratings for the first stressful situation, in a previous study, we found this scenario to have a similar mean stress rating compared to the stressor in Session 2.

In a further demonstration of the potential efficacy of the VR games, we also demonstrated that the training group was able to lower their breathing rate significantly more than the control group, demonstrating the utility of the boat game for training the slow-breathing technique.

We selected HRV (as signified by SDNN) as our primary index of physiological stress given its relation to respiratory sinus arrhythmia (Shaffer & Ginsberg, 2017). In line with best-standard recommendations, SDNN was corrected for HR (De Geus et al., 2019; Sacha, 2013). This is important since HR has a clear relationship with HRV: we would normally expect increases in HR to be associated with reductions in HRV. However, corrections of this kind are not universally found in psychophysiological studies, meaning that reports of altered HRV may be a consequence of altered HR rather than directly reflective of a true change in the physiological parameters that HRV is held to signify. The fact that we found increased HRV despite elevated HR in the stressor session therefore lends further weight to our observations of clear increases in HRV associated with respiratory control.

### 4.1 Limitations

There are several limitations to these studies that are worth noting and that future work may focus on. The high degree of dropout due to technical problems with the respiratory measurement setup, predominantly affecting Study 1, was unfortunate. This arose partly due to participant movement (affecting the respiration belt around their chest) and additionally a lack of appropriate respiratory band sizing for some of our participants. Loss of respiratory data was primarily due to movement and noise, although three participants were excluded from the analysis due to poor band-fitting, which could have introduced bias. Most data lost due to noise were in the baseline condition in Study 1, perhaps due to participants moving due to boredom while asked to sit quietly. While this problem was largely mitigated in Study 2, future work could replicate this study with larger samples of respiration data.

A second concern relates to the coding error that led to the respiration training biofeedback mechanism being the opposite of what was intended. In brief, while participants were instructed that lowering their HR would make the boat go faster, in fact, this made the boat go more slowly. Fortunately, speed changes would be unlikely to be perceived by participants, given they had no point of reference for the boat’s speed. The HR readout in the prow of the boat was unaffected, as were the verbal and visually presented respiratory training instructions. Given this, and the fact that the respiratory training did have a significant effect on ensuing performance in the stress task, we do not feel that this error has affected the key findings. Nonetheless, it is important to acknowledge the possibility.

An important, and seemingly paradoxical, observation is that in Study 2, despite the physiological changes, the training group reported the stressor experience in Session 2 as significantly more stressful than the control group. The increase in subjective stress of the training participants in Study 2 requires further exploration, as this may negatively affect potential clinical utility. One important difference between the two groups is that the training group was aware that their ability to apply the prior training was under scrutiny, meaning that the task was akin to a test. Future studies will examine the extent to which this may be a factor in determining levels of subjective stress and how these may be mitigated through experimental context, setup, and instructions. This raises questions about future experiments and whether changes can be made to the experimental procedure or the games themselves to promote less stress.

## 5 Conclusions

Biofeedback is a method of reducing stress and anxiety and a useful adjunctive treatment for established clinical approaches to anxiety, such as pharmacological interventions and psychotherapies. Biofeedback could also be used for patients who are currently on treatment waiting lists or those struggling to access standard care pathways. Given that recent research has demonstrated the utility VR has for psychological treatment, and as a tool for clinical practice and research (Bell et al., 2020; Valmaggia et al., 2016), we have sought to show that it is a convenient, acceptable, and safe method of generating a moderately stressful experience in VR and that this experience can provide a useful context for practising biofeedback regulation. We showed that VR facilitated a smooth transition from a training environment to a stressful environment, enabling a more immediate practice of a physiological regulation technique in a stressful environment. While the stress experience itself is evidently unrealistic, it was deliberately used in this study as a scenario that would cause stress in most participants. Future work could explore the development of more personalized stressors for patients, potentially combining elements of biofeedback with exposure therapy. Additionally, future work could examine whether regulation skills acquired in an unrealistic environment like the horror scenario described can be generalized into real-world stressful environments, and whether the skills are situation-specific or generally applicable.

Notwithstanding the limitations described above, our study points to the potential promise of VR in providing engaging, immersive convenient, and experimentally flexible experiences for testing and developing potentially valuable clinical tools. Furthermore, incorporation of gameplay into these experiences lends further potential, particularly in the pressing search for therapies that are attractive and accessible to a wide range of people, including those outside the clinical domain.

## Supplementary Material

Additional supporting information can be found online in the Supporting Information section at the end of this article.

Data S1

## Figures and Tables

**Table 1:** Differences between participants with and without respiration data.

	Ps with respiration data (*n* = 29)	Ps without respiration data (*n* = 18)
Age mean (SD)	25.8 (4.70)	25.2 (4.45)
%female	45%	55%
Trait anxiety mean (SD)	39.55 (9.18)	39.67 (10.03)
Baseline HR mean	77.09	80.51

## Data Availability

Data and code for these studies are available at https://github.com/lucied-w/.
