# Supplementary material for: Using a virtual reality game to train biofeedback‐based regulation under stress conditions
Source: Psychophysiology. 2024 Oct 9;62(1):e14705. doi: 10.1111/psyp.14705 (PMC7617308; doi:10.1111/psyp.14705)
Supplement: Supplementary file 1 — Data S1: [file PSYP-62-e14705-s001.docx]

**Supplementary information**

Study 1: Proof of Concept

*Training session*

During the training participants were asked to breathe slowly at a rate of 4 breaths per minute. This led to an increase in cvSDNN in the training condition. A repeated measures ANOVA found a significant effect of time, F(2, 86) = 17.30. Post-hoc paired t-tests revelaed there was a significant difference between baseline and training, T = -5.88, p < 0.0001, baseline and stressor, T= -4.65, p < 0.0001, but no significant difference between training and stressor.


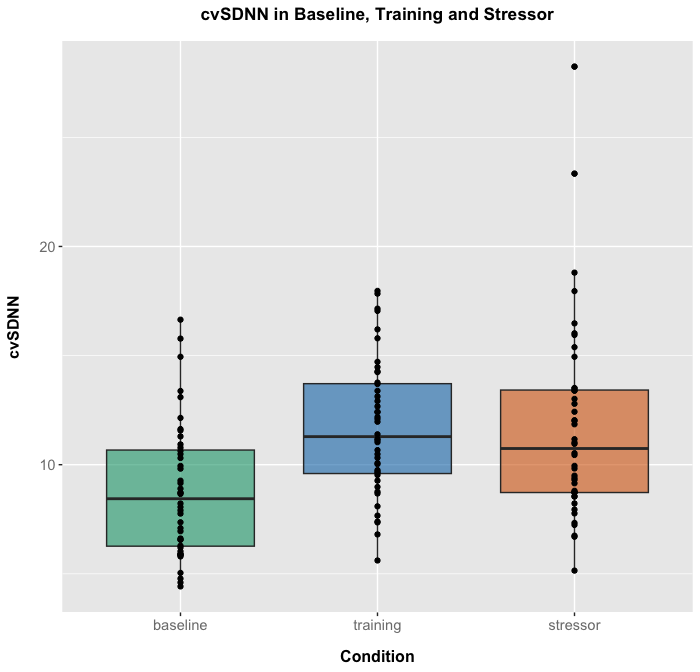


Fig. 1. cvSDNN in Baseline, Training and Stressor in Study 1.

*Adherence Scores*


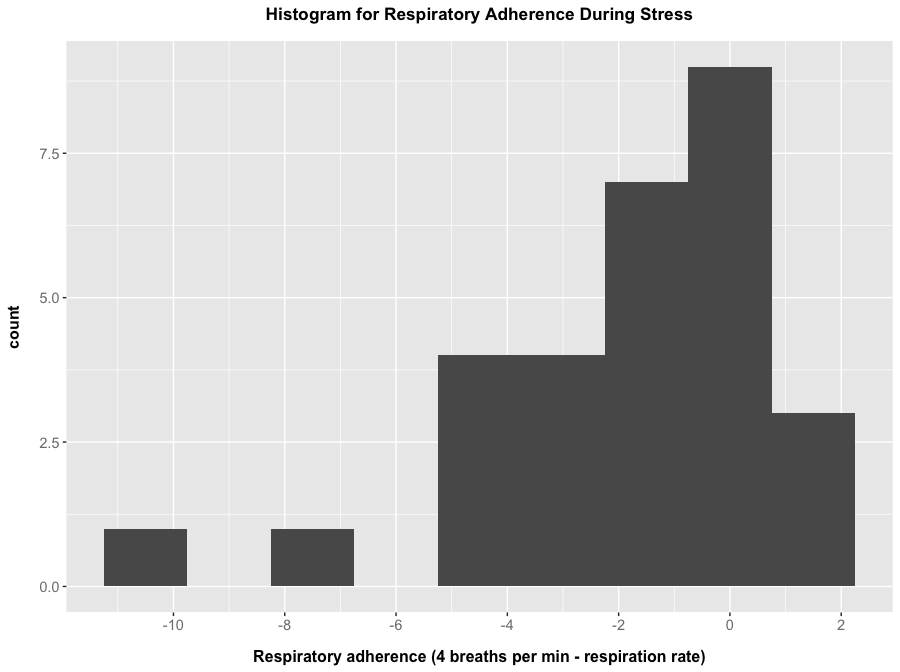


Fig 2. Histogram of respiratory adherence scores in Study 1.

Histogram of respiratory adherence scores during the stressor. Many participants were able to continue breathing at the trained rate of 4 breaths per minute, or close to it (up to 6 breaths per minute). 3 participants managed a breathing rate of 3 breaths per minute. Towards the left hand size of the histogram there are the participants who returned to a higher breathing rate, often close to their baseline level.

**Uncorrected SDNN Values**

Following the guidelines suggested by xxx we have included the main analyses from Study 1 and Study 2 with the uncorrected SDNN values (i.e., SDNN uncorrected for HR). The data for these analyses are also available on our github (see <https://github.com/lucied-w/biofeedback-concept-paper> and <https://github.com/lucied-w/two-arm-biofeedback> for details).

***Study 1***

SDNN baseline – stressor paired t-test

Uncorrected SDNN showed an increase between baseline and stressor. Mean SDNN at baseline was 70.18 compared to 85.20in the stressor, t(43) = - 3.100, p

= 0.003.

Correlation between SDNN and respiration

Correlations between baseline-normalised respiration and baseline-normalised SDNN did not show significant correlation, r(24) = 0.34, p = 0.086

***Study 2***

Control training group paired T-test in stressor

Baseline normalised SDNN did showed a significant difference, with the training group (21.15) showing higher SDNN than the control (-8.38), T= 2.77, p = 0.002.

Two-way repeated measures ANOVA

The two-way repeated measures ANOVA of the SDNN data showed a significant difference in group F(1, 50) = 8.96, p = 0.004, time F(1, 50) = 5.77, p = 0.02, and an interaction in group*time, F(1, 50) = 6.91, p = 0.011. Post-hoc analyses found that the dungeon biofeedback stressor had a higher mean SDNN (6.38) overall than the intruder scenario (-3.40), p = 0.002. The training group also had a higher SDNN (10.90) than the control group (-7.92), p < 0.001. The interaction effect demonstrated that the training group showed a higher SDNN in the biofeedback stressor over the intruder scenario when compared to the control group, p < 0.001.
